# P-1958. Impact of COVID-19 on Acute Diverticulitis: Evidence of United States Hospitals in 2020

**DOI:** 10.1093/ofid/ofae631.2117

**Published:** 2025-01-29

**Authors:** Thanathip Suenghataiphorn, Tuntanut Lohawatcharagul, Pojsakorn Danpanichkul, Jerapas Thongpiya, Natchaya Polpichai, Narathorn Kulthamrongsri, Ponphisudti Tangsirisatian, Nuttarak Sasipong, Phuuwadith Wattanachayakul

**Affiliations:** Griffin Hospital, Ansonia, Connecticut; Faculty of Medicine, Chulalongkorn University and King Chulalongkorn Memorial Hospital, Bangkok, Krung Thep, Thailand; Texas Tech University Health Science Center, Lubbock, Texas; Texas Tech University Health Science Center, Lubbock, Texas; Weiss Memorial Hospital, Chicago, Illinois; Mayo Clinic, Pheonix, Arizona; Faculty of Medicine, Chulalongkorn Hospital, Bangkok, Thailand, Bangkok, Krung Thep, Thailand; Faculty of Medicine, Chulalongkorn Hospital, Bangkok, Thailand, Bangkok, Krung Thep, Thailand; Albert Einstein Healthcare Network, Philadelphia, Pennsylvania

## Abstract

**Background:**

Acute diverticulitis, a prevalent gastrointestinal condition, frequently necessitates hospitalization and is associated with potential adverse in-hospital outcomes. Recent data showed the detrimental effects of COVID-19 infection on acute diverticulitis, particularly in patients with severe COVID-19 infections. However, limited information exists on the specific impacts of COVID-19 infection in patients hospitalized for acute diverticulitis.

**Methods:**

We analyzed the 2020 U.S. National Inpatient Sample (NIS) to investigate the effects of COVID-19 infection on cases primarily admitted due to acute diverticulitis. Participants aged 18 and above were identified using relevant ICD-10 CM codes. Adjusted odds ratios (aORs) for specified outcomes were calculated through multivariable logistic and linear regression analyses. The primary outcome was inpatient mortality, with secondary outcomes including system-based complications. Statistical significance was established at a P value of 0.05.

**Results:**

We identified 167,170 patients with a primary discharge diagnosis of acute pancreatitis. The mean age was 60.1 years; 56.8% were female. Caucasians accounted for the majority, followed by Hispanics. Of these, 0.90% (1,504/167,170) had a concurrent diagnosis of COVID-19 infection.

In a survey multivariable logistic and linear regression model adjusting for patient and hospital factors, COVID-19 infection was associated with higher in-hospital mortality (aOR 8.43; 95% CI 4.32, 16.42, P < 0.001), prolonged length of stay (β LOS 2.02; 95% CI 1.27, 2.77, P < 0.001) and increased hospitalization charge ( β charge 17,813.27; 95% CI 4,080, 31,546, P < 0.001), We observed non-significant, but increased, risks of shock, acute kidney injury (AKI), acute respiratory distress syndrome (ARDS), and mechanical ventilation in COVID-19 positive patients.

**Conclusion:**

In conclusion, our study revealed that COVID-19 infection is associated with higher in-hospital mortality, prolonged hospital stays, and increased hospitalization charges in patients diagnosed with acute diverticulitis. Future longitudinal studies are warranted to comprehensively assess the long-term health sequelae in this population.

**Disclosures:**

All Authors: No reported disclosures

